# Spinal Venous Engorgement Secondary to Congenital Absence of the Infrarenal Inferior Vena Cava Mimicking Cauda Equina Syndrome

**DOI:** 10.7759/cureus.94325

**Published:** 2025-10-11

**Authors:** Ahmed Mohammed

**Affiliations:** 1 Trauma and Orthopaedics, Lincoln County Hospital, Lincoln, GBR

**Keywords:** congenital ivc absence, epidural venous plexus, inferior vena cava anomaly, neurovascular mimic, spinal venous engorgement, suspected cauda equina syndrome, venous thrombosis

## Abstract

This case report contributes to the limited literature by describing a man in his early forties who presented with symptoms suggestive of cauda equina syndrome (CES). Initial investigations focused on identifying a possible spinal lesion as the underlying cause. However, further imaging and multidisciplinary team assessment revealed a rare vascular anomaly: agenesis of the infrarenal segment of the inferior vena cava (IVC), a condition with a distinct etiology that requires a fundamentally different approach to management.

## Introduction

Low back pain (LBP) is one of the most common presenting complaints in the United Kingdom and has placed a considerable burden on general practitioners (GPs) and orthopedic departments, particularly in settings where specialized spinal services are unavailable [1,2]. Owing to the high case volume, clinical attention is frequently directed toward excluding cauda equina syndrome (CES). However, this focus may serve as a diagnostic red herring, as several other conditions can present with symptoms that closely resemble CES [2,3]. One such condition is agenesis of the inferior vena cava (IVC).

The right-sided inferior vena cava (IVC) completes development by the eighth week of fetal growth. The infrahepatic segment of the IVC is derived from three paired embryonic veins: posterior cardinal, subcardinal, and supracardinal veins. IVC agenesis is a rare anomaly with an unclear pathophysiology. Some researchers suggest an acquired etiology due to intrauterine or perinatal thrombosis. In infrarenal IVC agenesis, collateral circulation forms via four principal pathways: gonadal, paravertebral (azygos and hemiazygos), hemorrhoidal (portal), and superficial abdominal venous systems [4].

Awareness of these alternative diagnoses is essential, as it enables timely and appropriate investigation, prevents unnecessary procedures, enhances patient safety, and reduces the financial burden on the National Health Service (NHS). In this case report, we describe a rare condition: agenesis of the inferior vena cava (IVC), specifically involving the infrarenal segment.

## Case presentation

A man in his early forties presented with acute, severe lumbar back pain radiating into both legs, more pronounced on the right side. He subsequently developed bilateral lower-limb weakness, numbness in the perineal and scrotal regions, and urinary retention. Constipation was reported, though there was no bowel incontinence. He also described episodes of leg weakness that resulted in a fall.

His past medical history included bilateral deep vein thromboses, for which he remained on long-term anticoagulation with rivaroxaban, a direct oral anticoagulant. Additional medications included mirtazapine, quinine sulfate, and morphine sulfate.

On examination, muscle power in the left lower limb was normal (L2-S1), whereas the right lower limb demonstrated power of 3/5 across the same myotomes. Sensory testing revealed a global reduction over the right lower limb. Tone was preserved bilaterally. Reflexes were diminished in the right leg but preserved on the left. Digital rectal examination demonstrated reduced anal tone. In addition, perineal and scrotal sensation was reduced.

**Figure 1:**
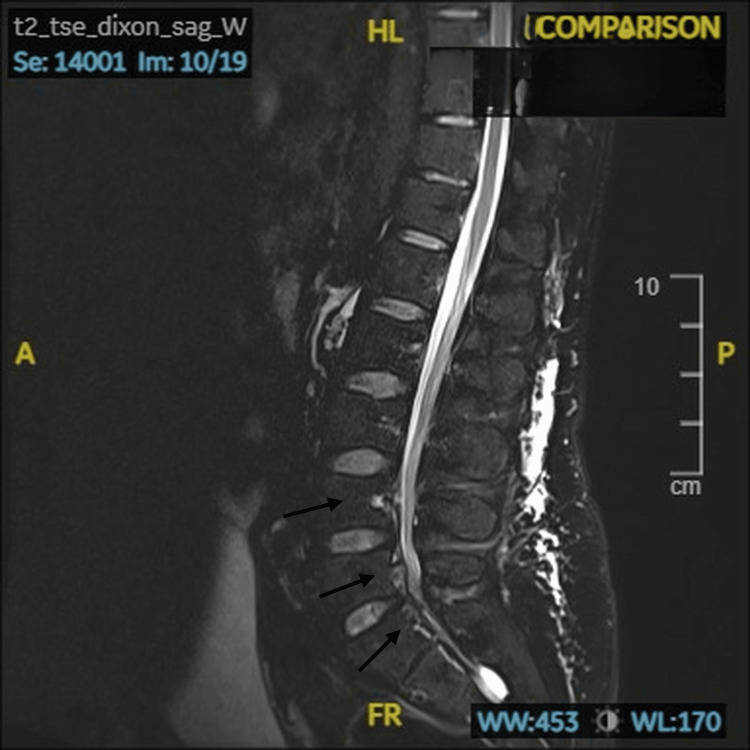
Prominent veins seen in the anterior epidural space at L4-L5 and S1 levels Sagittal view of MRI demonstrating prominent veins seen in the anterior epidural space at L4-L5 and S1 levels with the absence of disc herniation.

Magnetic resonance imaging of the spine excluded disc herniation, tumor, and abscess. Instead, there was marked engorgement of the epidural venous plexus compressing the thecal sac (Figure 1).

**Figure 2:**
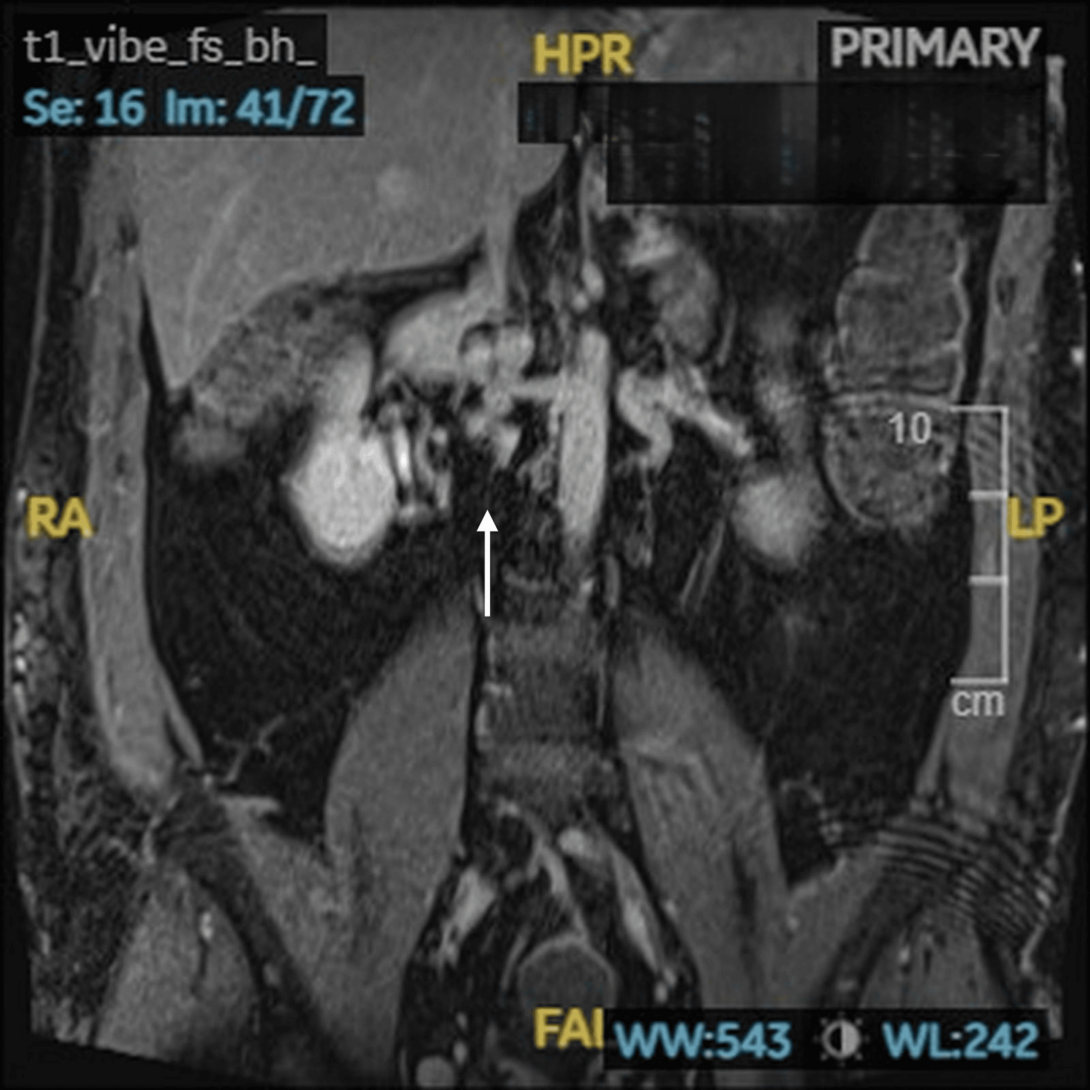
Nonvisualization of the infrarenal inferior vena cava. Coronal view of contrast-enhanced MRI of the inferior vena cava demonstrating nonvisualization of the infrarenal inferior vena cava.

Contrast-enhanced MRI of the inferior vena cava, which confirmed congenital absence of the infrarenal inferior vena cava (Figure 2).

These findings accounted both for the patient’s history of bilateral deep vein thromboses and for the acute presentation mimicking cauda equina syndrome.

The case was reviewed jointly by the spinal and vascular surgical teams. In the absence of a surgically amenable lesion, and given the risk of major bleeding from engorged collateral veins, conservative management was advised. The patient continued anticoagulation, received analgesia, and was enrolled in physiotherapy. He demonstrated gradual neurological recovery and was discharged with ongoing vascular surgery follow-up for long-term management.

## Discussion

Cauda equina syndrome (CES) is an uncommon but serious condition, most often resulting from a large lumbar disc herniation compressing the cauda equina nerve roots within the lower lumbar canal. If decompressive surgery is delayed, patients may suffer irreversible complications, including loss of bladder and bowel control and sexual function. Beyond patient outcomes, CES has also become a growing source of litigation, with significant legal and financial consequences for clinicians and healthcare institutions [5].

The inferior vena cava (IVC), formed by the union of the right and left common iliac veins, drains into the right atrium and receives tributaries from the lumbar, renal, right adrenal, right gonadal, and hepatic veins [6]. Agenesis of the IVC (IVCA) is a rare vascular anomaly, seen in only 0.0005%-1% of the population. Although uncommon, it is an important but often overlooked cause of deep venous thrombosis and pulmonary embolism in younger patients [7].

Notably, the clinical presentation of IVCA can occasionally mimic that of CES, as illustrated in this case. Venous congestion within the pelvis or lower limbs, together with the dependence on the IVC for lumbar venous drainage, may lead to elevated pressure around the lumbar spine. Such hemodynamic changes can produce neurological manifestations, including leg weakness, sensory disturbances, and bladder dysfunction, thereby posing a significant diagnostic challenge. Awareness of IVCA as a differential diagnosis is therefore essential to avoid misinterpretation, unnecessary surgical intervention, and delays in appropriate management.

Management of this condition is fundamentally different from that of true cauda equina syndrome. Surgical decompression is not only unnecessary but potentially harmful, given the reliance on collateral venous return and the high risk of hemorrhage. Instead, conservative measures, including anticoagulation, pain management, and physiotherapy, are appropriate. Multidisciplinary involvement of spinal and vascular teams is essential to ensure accurate diagnosis and safe management.

This case contributes to the limited body of literature describing congenital absence of the infrarenal inferior vena cava as a rare mimic of cauda equina syndrome. It emphasizes the need to maintain a broad differential diagnosis when evaluating acute neurological presentations, especially in young patients with unexplained venous thromboembolism.

## Conclusions

Congenital absence of the infrarenal inferior vena cava is a rare vascular anomaly that may mimic cauda equina syndrome by causing engorgement of the epidural venous plexus. Recognition of this condition on imaging is critical to avoid unnecessary and potentially harmful surgery. Conservative management, supported by multidisciplinary collaboration, can result in favorable outcomes.
